# Development of an Integrated Multifunctional Column for Rapid Pretreatment and Determination of Trichothecenes in Cereals and Feeds with HPLC-MS/MS

**DOI:** 10.3390/foods14091466

**Published:** 2025-04-23

**Authors:** Sisi Liu, Yu Wu, Tongtong Liu, Jin Ye, Li Li, Xiao Guan, Songxue Wang

**Affiliations:** 1 School of Health Science and Engineering, University of Shanghai for Science and Technology, Shanghai 200093, China; s15907925623@126.com (S.L.); gnxo@163.com (X.G.); 2 NFSRA Key Laboratory of Grain and Oil Quality and Safety, Academy of National Food and Strategic Reserves Administration, Beijing 100037, China; wyu@ags.ac.cn (Y.W.); wy13270353@163.com (T.L.); ll@ags.ac.cn (L.L.)

**Keywords:** multifunctional column, trichothecenes, mycotoxins, HPLC-MS/MS, cereals, feeds

## Abstract

The frequent detection of trichothecenes in grains highlights critical health risks to humans and animals. Based on the hybrid sorbent strategies, this study developed an innovative multifunctional column (ASAG563) integrating extraction, purification, and filtration to address limitations of existing methods, including cumbersome process, protracted duration, harmful to the environment, and significant matrix interference. Coupled with high-performance liquid chromatography–tandem mass spectrometry (HPLC-MS/MS), the ASAG563 column demonstrated superior recoveries (80.8–117.8%) and quantification limits (2.02~48.41 µg/kg) across cereals and feeds, with low relative standard deviations (<6.8%). Compared to commercial MFCs, the ASAG563 column simplified the process, reduced material consumption, saved 50% of analysis time, and effectively eliminated matrix effects. Analysis of 512 maize for feedstuff samples from Northeast China revealed significant contaminations with deoxynivalenol (DON) and its derivatives, emphasizing the necessity for enhanced regulatory measures. This novel integrated multifunctional pretreatment column presents a convenient, cost-effective, and eco-friendly solution for accurate TCT detection, significantly advancing analytical capabilities.

## 1. Introduction

Trichothecenes (TCTs) are mycotoxins produced by the Fusarium genus. Based on their chemical structure and fungal producers, they are broadly categorized into four types: A, B, C, and D. Type A and Type B TCTs are commonly contaminated in cereals and feeds, with Type A primarily including T-2 toxin and HT-2 toxin [1], while Type B is mainly represented by deoxynivalenol (DON) and its derivatives, posing significant threats to animal and human health [2,3]. In recent years, high-performance liquid chromatography–tandem mass spectrometry (HPLC-MS/MS) has become the preferred method for detecting TCTs due to its excellent selectivity, high sensitivity, and accuracy [4,5] because the complexity of cereal and feed matrices can easily lead to matrix effects, which not only impact the accuracy and stability of quantitative analysis but may also cause column blockage [6,7,8,9]. Therefore, pretreatment methods to reduce or eliminate matrix interference are crucial for achieving high sensitivity and precise detection of TCTs.

Currently, the mainstream pretreatment techniques for TCTs include Immunoaffinity Columns (IAC), QuEChERS (Quick, Easy, Cheap, Effective, Rugged, Safe), Solid Phase Extraction (SPE) and Multifunctional Purification Columns (MFC). The Immunoaffinity Column (IAC) technology is based on immunological principles, achieving efficient enrichment and purification of target compounds through specific adsorption of antigens and antibodies [10,11,12]. Although IAC technology possesses high selectivity, high enrichment efficiency, and good selectivity, its application is limited by the availability of specific antibodies, and it is relatively costly and complicated to operate. QuEChERS technology, as a broad-spectrum rapid pretreatment method, removes impurities by the interaction between the adsorbents and the impurities in the sample matrix [13,14,15,16]. However, existing QuEChERS methods exhibit deficiencies in purifying TCTs, and the operational steps are cumbersome, requiring multiple centrifugations, filtrations, and liquid transfers, resulting in high consumption of materials, time and labor costs, and significant environmental impact. MFC technology is based on chromatographic principles, which involve selecting appropriate sorbents to adsorb impurities from the extract [17]. MFC can effectively eliminate interfering substances such as lipids, pigments, and proteins [18,19]; however, its applicability is constrained by the choice of sorbents. It also needs multiple liquid transfers, centrifugation, and filtration, which can lead to quantification errors and is not environmentally friendly [20,21]. Many pretreatment methods have been developed using new adsorbents, both of which can effectively extract various mycotoxins from food [22,23,24]. However, the complex synthesis of these materials leads to significantly increased costs and is time-consuming. Considering the limitations of existing technologies, there is an urgent need to develop more convenient, cost-efficient, and eco-friendly pretreatment methods for TCTs to enable effective detection in complex matrices.

Hence, this study aimed to develop an integrated multifunctional column for the simultaneous extraction and determination of seven trichothecenes in maize for feedstuff. Firstly, we evaluated the adsorption performance and impurity removal capacity of different sorbents, filters, and sieve plates for TCTs. Then, we optimized the hybrid sorbents and selected the one with the best purification effect to design a novel column. Moreover, the accuracy and purification effect of the new column was systematically evaluated and verified according to the EU 2023/2782 [25] and SANTE 11312/2021 [26] standards. Finally, the novel integrated purification column was applied to pretreat the maize for feedstuff samples from Northeast China to analyze the contamination of TCTs.

## 2. Materials and Methods

### 2.1. Materials and Reagents

The materials used included the following: formic acid (98%), acetonitrile (ACN), acetic acid (≥99.7%), and ammonium acetate (LC-MS grade, Thermo Fisher Scientific, (Waltham, MA, USA); various types of sorbents, including C8, hydrophilic–lipophilic balance (HLB), mixed anion exchange (MAX), C18 alkyl (C18-AX), C18 cartridge (C18-CX), mixed cation exchange (MCX), octadecylsilyl (C18), graphitized carbon black (GCB), primary secondary amine (PSA), and polymeric material (POLY) (Anybond, Tianjin, China); Romer MFC 227 (ROMER International Trade (Beijing) Co., Ltd., Beijing, China); Pribolab MFC 227 (Pribolab Biotech, Qingdao, China); 0.2 μm PTFE filter connected with a syringe (PALL, Port Washington, NY, USA); pipettes (100–1000 μL, Eppendorf, Hamburg, Germany); and ultrapure water. Blank maize, blank Distillers Dried Grains with Solubles (DDGS), and certified reference material for deoxynivalenol in maize flour (GBW(E)100808, Academy of National Food and Strategic Reserves Administration, Beijing, China) were also used.

### 2.2. Standards

Standard solutions included nivalenol (NIV), deoxynivalenol (DON), 3-acetyl-deoxynivalenol (3-AcDON), 15-acetyl-deoxynivalenol (15-AcDON), deoxynivalenol-3-glucoside (DON-3G), T-2 toxin (T-2), HT-2 toxin (HT-2), aflatoxin B1 (AFB1), aflatoxin B2 (AFB2), aflatoxin G1 (AFG1), aflatoxin G2 (AFG2), fumonisin B1 (FB1), fumonisin B2 (FB2), fumonisin B3 (FB3), zearalenone (ZEN), ochratoxin A (OTA), and sterigmatocystin (ST) (Pribolab Biotech, Qingdao, China). Five stable isotope-labeled standard solutions of 13C-labeled NIV, DON, DON-3G, 3-AcDON, 15-AcDON, T-2, and HT-2 were all from Romer International Trading (Beijing, China).

### 2.3. Preparation of Solutions

The mixed standard solution was obtained by mixing individual stock solutions (μg/L) in ACN; detailed concentrations (C) are shown in Table S1. The mixed 13C-labeled working solution (μg/L) was obtained by mixing an appropriate volume of single 13C-labeled standards (μg/L); detailed concentrations (C) are shown in Table S2. All solutions were stored at −20 °C for subsequent use.

### 2.4. Instruments and Equipment

The instruments and equipment used included QTRAP^®^6500 + LC-MS/MS System (SCIEX, Singapore; Framingham, MA, USA) equipped with a CORTECS UPLC C18 column (100 mm × 2.1 mm, 1.6 μm; Waters, Milford, MA, USA); 5424R and 5810R high-speed refrigerated centrifuges (Eppendorf, Hamburg, Germany); ultrapure water system (PURELAB Chorus, ELGA LabWater, Lane End, UK); and multi-tube vortex mixer (VX-IV, SCDEALL, Beijing, China).

### 2.5. Hybrid Sorbents Optimization

Ten adsorbent materials (C8, HLB, MAX, MCX, C18, GCB, PSA, C18-AX, C18-CX, and POLY), each weighing 100 mg, were initially screened to assess their purification efficiency. Based on performance, four hybrid adsorbent groups (Groups 1–4) were formulated and systematically evaluated for their recoveries and matrix effects toward 17 mycotoxins (listed in Table S1) spiked in DDGS. The construction of hybrid sorbents was prepared as follows: Accurate weighing 200 mg of C18 and 250 mg of MAX were taken separately to prepare the first group of hybrid sorbents (Group 1). Accurate weighing 200 mg C18, 250 mg MAX, and 100 mg MCX were taken separately to prepare the second group of hybrid sorbents (Group 2). Accurate weighing 100 mg C18, 250 mg MAX, 100 mg MCX, and 50 mg POLY were taken separately to prepare the second group of hybrid sorbents (Group 3). Accurate weighing 100 mg C18, 250 mg MAX, 100 mg MCX, 50 mg POLY, and 50 mg GCB were taken separately to prepare the second group of hybrid sorbents (Group 4). All group hybrid sorbents were mixed within a column and stored at 25 °C for subsequent use.

### 2.6. Sample Collection

A total of 512 maize for feedstuff samples were collected from Northeast China. The collection process employed placement technology based on satellite remote sensing [27] in conjunction with grid sampling to gather maize kernels from various regions. Using a sampling spear, we took multiple-point samples from each bag (ca. 50–100 kg) at three different layers: low, medium, and high. Five sampling points were collected from each layer. The multiple subsamples (ca. 500 g) were thoroughly mixed before being processed through the coning and quartering method to produce a final representative sample also weighing 500 g. Subsequently, each representative sample was ground into a fine powder and passed through a 0.5 mm mesh screen. The sieved powder samples were then placed into sealed bags and stored at a temperature of 4 °C until further analysis could be conducted.

### 2.7. Sample Preparation

An amount of 1.0 g of the powdered sample was accurately weighed and placed in a novel integrated multifunctional column (ASAG563). An amount of 4 mL of extraction solvent (acetonitrile/water = 70:30, *v*/*v*) was added, and then the column with the cap was occluded and sealed for 21 min. The caps were opened to collect 1.5 mL of the eluate into a 2 mL centrifuge tube. An amount of 500 μL of the eluent was taken and diluted with 500 μL of ultrapure water. Next, 180 μL of the diluted solution was taken, and 20 μL of the internal standard solution was added. It was mixed thoroughly before proceeding with the analysis.

### 2.8. LC-MS/MS Analysis

Samples were analyzed using a QTRAP^®^ 6500 + LC-MS/MS system, with a CORTECS™ UPLC C18 column (100 mm × 2.1 mm, 1.6 μm) employed for chromatography. The column temperature was maintained at 40 °C, and the injection volume was set at 2 μL. Mobile phase A consisted of methanol, while mobile phase B was an aqueous solution containing 0.1% (*v*/*v*) formic acid and 1 mmol/L ammonium acetate, with a flow rate of 0.3 mL/min. The gradient elution program was as follows: from 0 to 2 min, 90% B; at 3 min, 80% B; at 4 min, 79% B; from 5 to 7 min, 74% B; at 8 min, 50% B; at 11 min, 48% B; from 12 to 15 min, 5% B; and from 15.1 to 18 min, 10% B.

The mass spectrometry conditions were consistent with those established in previous studies by our research team [28]. The ion source was set to Tube Spray, with the collision-induced dissociation energy set to CAD medium. Ion Source Gas 1 and Ion Source Gas 2 were both set to 55 psi, while the curtain gas (CUR) was set to 30 psi. The source temperature (TEM) was maintained at 350 °C, and the ion spray voltage (IS) was set to +4500/−4500 V. The scan time was 0.5 s. The mass spectrometry conditions for the 17 mycotoxins are provided in Table S3.

### 2.9. Data Analysis

The raw mass spectrometry data were analyzed using Analyst Software 1.7.1 (SCIEX, Singapore; Framingham, MA, USA). Data analysis, including the calculation of recoveries, matrix effects, and relative standard deviations, was performed using Microsoft Excel 2021 (Microsoft Corporation, Redmond, WA, USA). All result graphs and correlation analyses were generated using OriginPro 2024 software (Origin Lab, Hampton, MA, USA). The results are presented in the corresponding graphs.

### 2.10. Method Validation

Method validation followed the SANTE 11312/2021 [26], EU 2023/2782 [25], and EU No. 519/2014 [29]. The recovery, matrix effect, linearity, limits of quantification (LOQ), accuracy, and precision were systematically investigated using spiked samples in three concentration levels. Quantification was conducted using a stable isotope internal standard calibration method. The recovery (REC) and matrix effect (ME) were calculated using the following formulas:

(1) $$REC=\frac{{Ratio}_{sample}}{{Ratio}_{std}}\times 100\%$$

where Ratio_sample_ represents the peak area ratio of the analyte to its internal standard in the sample, and Ratio_std_ represents the ratio of the analyte peak area to the internal standard peak area. The matrix effects (ME) were calculated by the area ratio of standard in matrix extract (Area_std_ in matrix extract) and standard in solvent (Area_std_ in solvent).

(2) $$ME=(\frac{{Area}_{std\;in\;matrix}}{{Area}_{std\;in\;solvent}}-1)\times 100\%$$

## 3. Results and Discussion

### 3.1. Selection, Optimization, and Combination of Sorbents

#### 3.1.1. Construction of a Database for the Purification Capacity of Individual Sorbent

**Figure 1:**
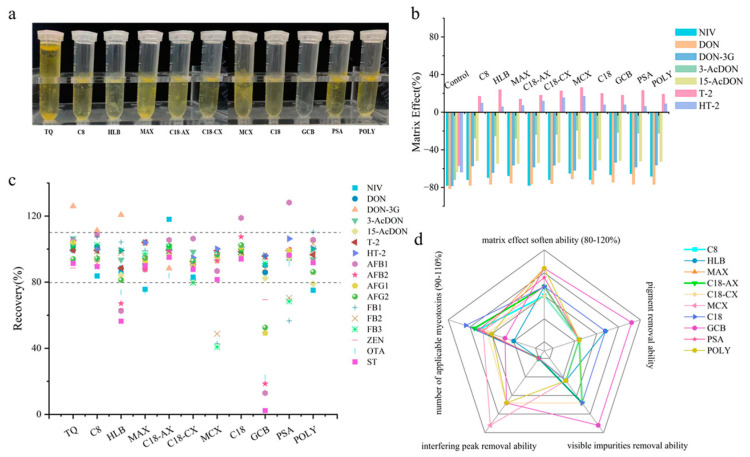
Investigation of the purification ability of sorbents: (**a**) photographs of purified samples; (**b**) matrix effects; (**c**) recoveries (The dotted line indicates the satisfied recovery range); (**d**) radar chart of comprehensive performance of sorbents.

Mycotoxins have complex structures and significant differences in physicochemical properties; therefore, it was important to choose an effective sorbent for purification in complex matrices. To investigate the sorption capacity and selectivity of different sorbents, an experiment was conducted using DDGS samples spiked at a medium concentration level, which represents a typical complex matrix in feed. The selectivity and sorption capacity for impurities of seven common and three newly modified sorbents (C8, HLB, MAX, MCX, C18, GCB, PSA, C18-AX, C18-CX, POLY; see Table S4 for their properties) were co-evaluated for seven TCTs (NIV, DON, DON-3G,3-AcDON, 15-AcDON, T-2, and HT-2) and ten other mycotoxins (AFB_1_, AFB_2_, AFG_1_, AFG_2_, FB_1_, FB_2_, FB_3_, ZEN, OTA, and ST). The results for DDGS purified with different sorbents compared to the unpurified sample (Control) are shown in Figure 1.

As observed in the total ion chromatogram (TIC) shown in Figure S1, both C18-CX and MCX sorbents exhibited notable sorption capacity for the interfering peaks at retention times of 6.9 min and 9.5 min. MCX demonstrates superior removal efficiency compared to C18-CX. This can be attributed to the cation exchange property of both MCX and C18-CX, which provide effective adsorption capabilities for basic and non-polar impurities. GCB showed remarkable adsorption efficiency for the interfering peak at a retention time of 11.7 min. The extracted solution exhibited the lightest color after purification, indicating that GCB has a good sorption ability for pigments and visible impurities [30], as shown in Figure 1a. This effectiveness can be linked to the six-membered aromatic ring of GCB, which exhibits strong adsorption capabilities for planar pigments. Furthermore, HLB, C18-CX, and C18 also demonstrated soft purification ability on pigments and lipids, and the effect of sorbents on matrix effects is different for TCTs, according to Figure 1b. However, no single adsorbent was able to soften the matrix effects for all mycotoxins. Figure 1c indicated that C8, C18-AX, C18-CX, and C18 sorbents achieved good recoveries for 17 mycotoxins (79.7–118.9%). However, they had weaker capabilities in removing pigments and impurities, as illustrated in Figure 1d. Other sorbents, such as GCB, demonstrated significant purification ability but exhibited high adsorption for aflatoxins, resulting in recoveries below 50%.

The radar chart in Figure 1d illustrates the comprehensive performance of ten types of sorbents based on five key performance indicators: removal of matrix effects, pigments, visible impurities, mass spectrometry interference peaks, and applicable mycotoxins. The performance of the sorbents was significantly different across these five key indicators. No single sorbent demonstrated satisfied performance, highlighting the limitations of individual sorbents in meeting the diverse purification requirements for various matrices. Therefore, this study has established a database for the purification capacity of individual sorbent for 17 mycotoxins, which includes information on the functional groups, properties, matrix effects, applicable mycotoxins, and removal ability of pigments, visible impurities, and mass spectrometry interference peaks (see Table S4). The database will be continually updated to include new sorbents and evaluate their related performance, aiming for precise purification of different mycotoxins in various matrices. For instance, GCB sorbents are recommended for matrices with heavy pigments due to their strong adsorption capacity for planar pigments. To minimize interference from acidic impurities when detecting mycotoxins other than fumonisins, MCX sorbents are suggested. For simultaneous detection of multiple mycotoxins, sorbents such as C18 and POLY, which exhibit low affinity for mycotoxins, are recommended to ensure high recoveries while reducing interference from impurities. This diversified sorbent selection strategy significantly enhances the sensitivity and accuracy of mycotoxin detection, addressing the purification needs of diverse matrices and target compounds.

#### 3.1.2. Optimization of Hybrid Sorbents

Based on the database for the purification capacity of individual sorbents, this study compared a series of sorbents with exceptional purification ability and no adsorption of target TCTs. After a thorough evaluation of the chemical properties and adsorption characteristics of the sorbents, we selected the following sorbents with different chemical properties to construct an efficient purification system: (1) GCB sorbent, which exhibits excellent capabilities for pigment removal; (2) C18 and POLY sorbents, which have high adsorption affinity for lipids, particularly POLY, which is particularly effective in removing interference peaks at a retention time of 11.72 min; (3) MAX sorbent, which has a good adsorption ability for acidic impurities such as fatty acids; (4) MCX sorbent, which exhibits remarkable removal effectiveness for basic and non-polar impurities and effectively removes interference peaks with retention times of 6.9 min and 9.54 min.

**Figure 2:**
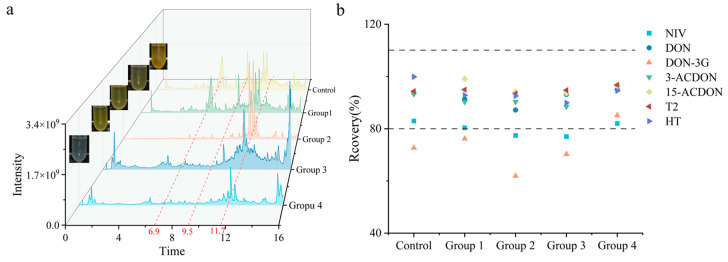
Purification efficiency of hybrid sorbents: (**a**) TIC (the dotted lines at 6.9, 9.54 and 11.7 min indicate the retention times of the interference peaks); (**b**) recoveries of seven TCTs. (Group 1: 200 mg C18 + 250 mg MAX; Group 2: 200 mg C18 + 250 mg MAX + 100 mg MCX; Group 3: 100 mg C18 + 250 mg MAX + 100 mg MCX + 50 mg POL; Group 4: 100 mg C18 + 250 mg MAX + 100 mg MCX + 50 mg POLY + 50 mg GCB, dashed range indicates satisfied recovery range).

Considering the adsorption capacities of the sorbents, we designed four groups of hybrid sorbents (described in Section 2.5) based on the selected signal sorbents. The purification abilities of the four hybrid sorbents were systematically evaluated using spiked DDGS. The TICs for different hybrid sorbents (Figure 2a) showed that the Group 4 hybrid sorbent exhibited significant effectiveness in removing interference peaks with retention times of 6.9 min, 9.54 min, and 11.72 min. Furthermore, Group 4 showed the most notable performance in the purification of pigments and impurities. This phenomenon may be attributed to several factors. First, C18 and POLY adsorb lipid interferences through hydrophobic interactions, while the graphitized carbon structure of GCB can adsorb pigments [31]. As a result, the elution of the sample is purer than that of the other groups. Additionally, the strong anion exchange properties of MAX and the strong cation exchange properties of MCX work together to adsorb acidic and basic impurities present in the sample [32]. The combined effects of these components contribute to the fourth group of composite fillers, demonstrating the best purification results. Recovery shown in Figure 2b indicated that Group 4 achieved satisfied recoveries for the seven TCTs within the acceptable range from 70% to 120% [25,26], significantly improving the selectivity of seven TCTs, detection levels, and global analytical performance. Hybrid sorbents from Groups 1, 2, and 3 showed lower recovery of DON-3G. Ultimately, Group 4 hybrid sorbent was selected as the sorbent for a dedicated integrated column of TCTs.

### 3.2. Design, Construction, and Optimization of Multifunctional Pretreatment Column

#### 3.2.1. Selection of Sieve Plate and Filter Membrane

The sieve plate is a key component of MFC, serving the dual functions of securing the packing material and controlling the flow rate. An ideal sieve plate material should possess the following characteristics: low water absorption, high chemical stability, resistance to dissolution and corrosion from common laboratory solvents, and no adsorption to the target analytes. After extensive research, we found that polyethylene, due to its excellent inertness and chemical stability, is a potential candidate material; common laboratory solvents do not dissolve or corrode polyethylene. Furthermore, ultra-high molecular weight polyethylene (UHMWPE) has high purity and a uniform pore size distribution, resulting in exceptional filtration performance [33,34]. UHMWPE is subjected to reflux purification with organic solvents to ensure that no impurities are released during use. We investigated the adsorption characteristics of polyethylene sieve plates for seven TCTs, and the results showed that the recoveries for the polyethylene sieve plates ranged from 89.5% to 99.3% (Figure S2), meeting international standards [25,26], indicating almost no adsorption to these seven TCTs. Therefore, we ultimately selected UHMWPE as the sieve plate material. For pore size design, we set the pore size of the upper sieve plate to 20 µm, which is smaller than the particle size of the packing material, effectively preventing the packing from spilling while allowing small particulate impurities to pass through, thus avoiding blockage of the sieve plate. The pore size of the lower sieve plate is designed to be 5 µm, which maximizes the interception of impurity particles while ensuring the permeability of the lower filter membrane to prevent clogging. Through this carefully designed sieve plate structure, we ensured the efficiency and reliability of the solid-phase extraction column during the extraction process, providing assurance for subsequent analytical work.

Filtration is an important step in sample preparation, playing a crucial role in protecting chromatographic systems, extending the lifespan of chromatographic columns, and improving data accuracy [35]. Therefore, prior to sample analysis, it is common to use syringe filters to filter samples, which requires additional syringes and is time-consuming and labor-intensive. To address this issue, we employed ultrasonic welding technology to integrate the filter membrane into the purification column, achieving a one-step purification and filtration process. When selecting a filter membrane, two key requirements should be considered: it must be compatible with acid, alkaline, and organic solvents present in the sample without adsorbing the target analytes [36]; the pore size should effectively intercept impurities while allowing the target analytes in the sample to pass through smoothly [34,37]. Polytetrafluoroethylene (PTFE) membranes have the broadest chemical compatibility, making them suitable for filtering various organic solvents, with good resistance to acids, alkaline and organic solvents [38,39]. Considering the particle size range of existing chromatographic columns typically varies from 1.7 to 3.5 µm, we conducted a detailed investigation and found that PTFE membranes showed no significant adsorption of seven types of TCTs, including DON, with recoveries ranging from 89.5% to 99.3% (Supplementary Figure S2), this may be attributed to the solvent compatibility of PTFE [34], and PTFE’s 0.2 μm pore size aligns with HPLC column particle sizes (1.7–3.5 μm), preventing clogging while allowing analyte passage. Based on these considerations, we ultimately selected a 0.2 µm PTFE membrane as the medium for sample filtration.

#### 3.2.2. Design of an Integrated Multifunctional Pretreatment Column

To minimize analyst manipulation, this study designed a novel integrated multifunction pretreatment column that integrates extraction, purification, and filtration, significantly simplifying the operational workflow and reducing errors and experimental discrepancies caused by multiple transfers and centrifugation steps. In selecting the column material, ultra-high molecular weight polyethylene (UHMWPP) was chosen as the preferred option due to its excellent chemical inertness and stability. UHMWPP has a very high tolerance to commonly used laboratory solvents, and its high purity characteristics ensure that no impurities leach out of the column during use. Additionally, UHMWPP was used for column development due to its favorable processing properties, enabling simple and efficient mold injection.

**Figure 3:**
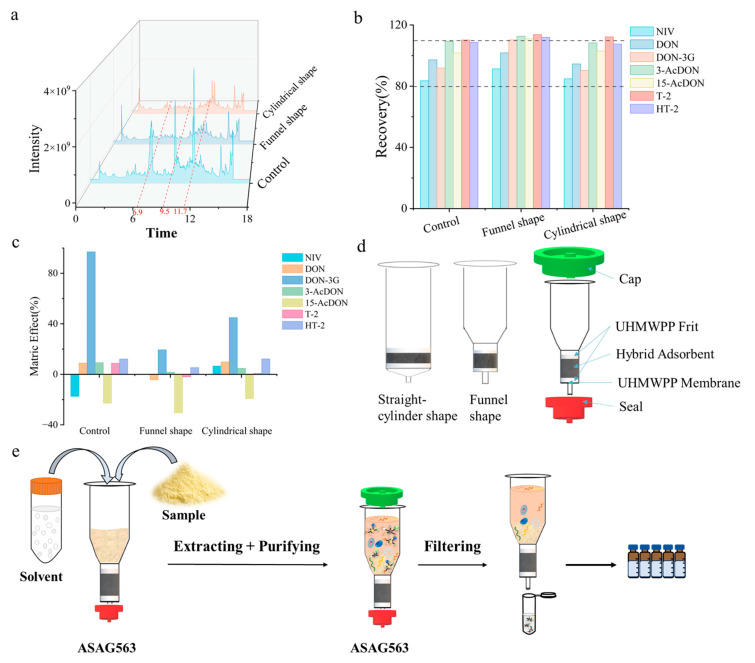
Evaluation of straight cylindrical shape and funnel-shaped columns by spiked DDGS: (**a**) TIC (the dotted lines at 6.9, 9.54 and 11.7 min indicate the retention times of the interference peaks); (**b**) recoveries (dashed range indicates satisfied recovery range); (**c**) matrix effects; (**d**) design diagrams of straight cylinder shape, funnel shape columns, and ASAG563; (**e**) flowchart of the pretreatment process using ASAG563.

For column design, we conducted a systematic evaluation of cylindrical and funnel-shaped columns, particularly focusing on the recoveries of TCTs and the absorption of impurities in complex matrices. The results indicate that the funnel-shaped column demonstrated superior interference removal efficiency, recovery, and soft matrix effects compared to the large-diameter cylindrical column (Figure 3a–c). This phenomenon can be attributed to the chromatographic efficiency of the elongated column, which helps the extraction solution interact more effectively with the packing material, thereby enhancing purification efficiency. To prevent leakage during extraction, we incorporated cap and seal into the design, achieving a single-step process for extraction, purification, and filtration, ultimately finalizing the design scheme of the column (Figure 3d). This column is filled with the optimized Group 4 hybrid sorbents between two sieves, allowing the sample to be purified through the column by gravity. This design enables the newly developed purification column (ASAG563) to integrate extraction, purification, and filtration without the need for multiple centrifugation steps and liquid transfers (Figure 3e).

### 3.3. Optimization of Extraction and Elution Solvents

**Figure 4:**
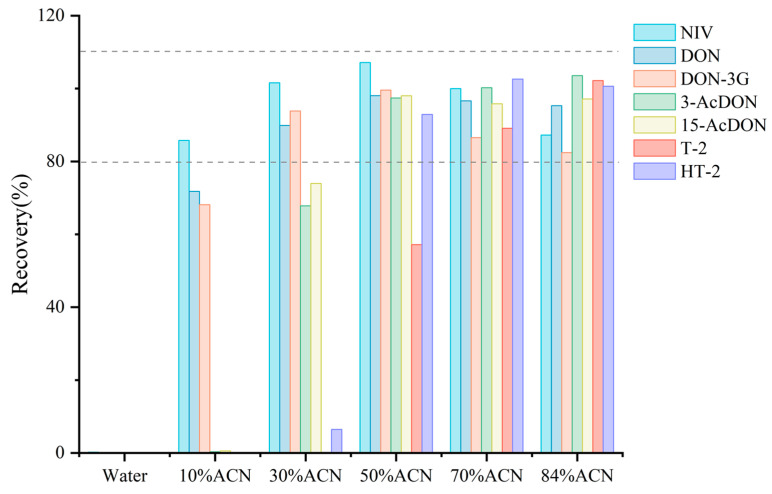
Recovery of TCTs with different solvents (Dashed range indicates satisfied recovery range).

To evaluate the adsorption and elution efficiency of the novel purification small column for TCTs under different solvent conditions, we experimentally investigated the recoveries for spiked DDGS using various proportions of acetonitrile (10%, 30%, 50%, 70%) as extraction and elution solvents. The experimental results are shown in Figure 4. As the proportion of acetonitrile increased, the elution capacity markedly improved, which resulted in higher recoveries for the TCTs. At a 30% acetonitrile proportion, only the recoveries for NIV, DON, and DON-3G ranged between 85% and 110%. Increasing the acetonitrile proportion to 50% resulted in recoveries for NIV, DON, DON-3G, 3-AcDON, 15-AcDON, and T-2, all falling within the same range of 85% to 110%, except HT-2 (<60%). At 70% acetonitrile, the recoveries for all seven TCTs were within the acceptable range of 80% to 110%, satisfying experimental requirements. When the acetonitrile proportion was further increased to 84%, recoveries for all seven TCTs remained between 80% and 110%; however, NIV and DON-3G exhibited slight decreases compared to the recoveries observed at 70% acetonitrile. Based on these findings and the reduction in organic solvent usage, 70% acetonitrile was selected as the extraction and elution solvent.

### 3.4. Analytical Performance of ASAG563

To evaluate the performance of the ASAG563 purification column, we conducted stringent validation experiments aimed at ensuring its reliability for detecting TCTs. The background of the purification column was assessed by randomly selecting three ASAG563 columns, each of which was passed through 1.5 mL of the extraction solution (acetonitrile/water = 70:30, *v*/*v*) for instrumental analysis, which indicated that the ASAG563 columns are free from background interference as shown in Table 1. To evaluate the column recovery, we randomly selected three additional ASAG563 purification columns, which were also passed through 1.5 mL of a medium-concentration solution containing seven TCTs. The recoveries for DON and its derivatives, T-2 and HT-2, using the ASAG563 purification column ranged from 88.4% to 100.8%, with relative standard deviation (%RSD) values of less than 5.5%, meet international standards [25,26]. These results indicate that the ASAG563 purification column meets the standard requirements for routine analysis.

### 3.5. Method Validation

In order to systematically evaluate the applicability and accuracy of the ASAG563 purification column for detecting TCTs in cereal and feed matrices, this study examined the linearity, limits of quantification (LOQ), and recoveries at low, medium, and high spiked concentration levels (n = 3) for two matrices: maize for feedstuff and DDGS. The results are shown in Table S5, indicating that the correlation coefficients for the linearity of the seven TCTs were all greater than 0.9990, the LOQ ranges for these TCTs in maize and DDGS matrices were found to be between 2.02 and 48.41 μg/kg. In the maize matrix, seven TCTs showed the soft matrix effect (5.3–6.5%). In DDGS samples, all the TCTs showed a soft matrix effect except DON-3G (32.8%), demonstrating a medium matrix effect. Most TCTs showed a matrix effect of less than 25%, which satisfies the acceptable matrix effect range set according to SWGTOX guidelines [40]. In maize samples, the recovery for all TCTs was observed within the range of 92.2–117.8%, with both intra-day and inter-day relative standard deviation (RSD_r_ and RSD_R_) values being less than 6.8%. In the DDGS matrix, recoveries ranged from 78.9% to 112.0% [25,26], with RSD_r_ and RSD_R_ values below 4.8% (shown in Table 2). In summary, the ASAG563 purification column is suitable for detecting monomeric TCTs in cereals.

To further validate the accuracy of the ASAG563 purification column in detecting TCTs in actual contaminated samples, this study employed the ASAG563 purification column to pre-process certified reference material of DON in maize for feedstuff (n = 6). The detection results are provided in Table S6, which indicated that the assigned values for DON were within the assignment range of the reference materials (860–1120 µg/kg), with an RSD of 2.3%. Thus, the ASAG563 purification column is suitable for the analysis of TCTs in contaminated cereal and feed samples.

### 3.6. Comparison with Commercial MFC

**Figure 5:**
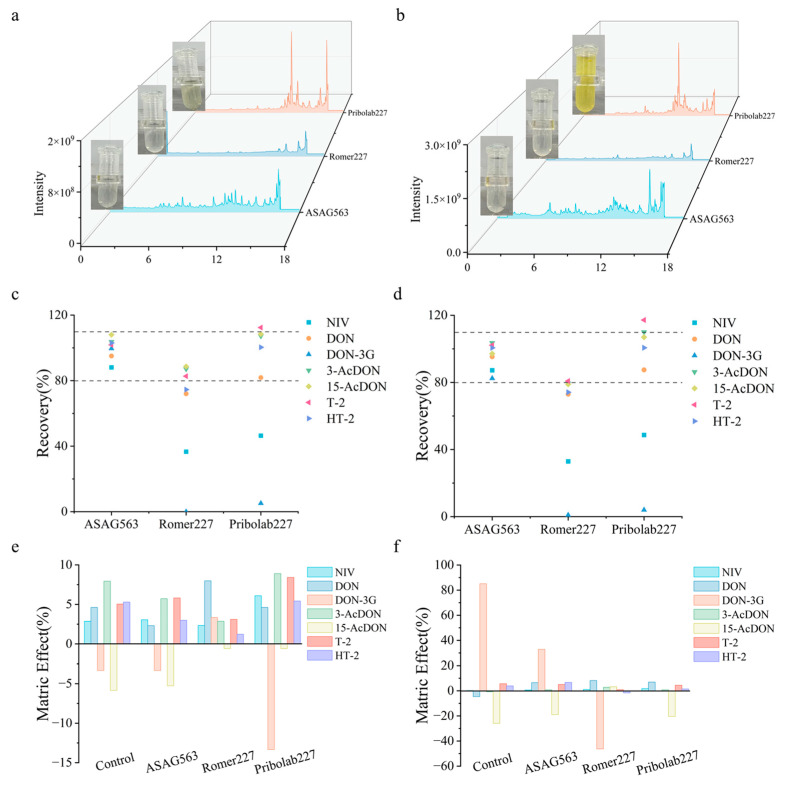
Comparison of purification efficiencies: (**a**) maize matrix TIC profile; (**b**) DDGS matrix TIC profile; (**c**) recoveries in maize; (**d**) recoveries in DDGS; (**e**) matrix effects in maize; (**f**) matrix effects in DDGS. Dashed range indicates acceptable range.

In this study, the purification performance of the ASAG563 column was compared to that of the commercial products Romer227 and Pribolab227 using spiked samples at medium concentration levels in maize and DDGS matrices. The experimental results are shown in Figure 5. The TIC results and the images of purified solutions (Figure 5a,b) indicate that the ASAG563 purification column was highly effective in removing pigments and impurities, comparable to the performance of the Romer227 purification column but clearly superior to Pribolab227. The recoveries of seven TCTs after purification with the ASAG563 column ranged from 85% to 110% for the spiked maize and DDGS samples. In contrast, the commercial products Romer227 and Pribolab227 exhibited poor recoveries for NIV and DON-3G, both of which fell below 50% (Figure 5c,d). The matrix effects of the commercial purification columns Romer227, Pribolab227, and ASAG563 ranged from −46.27% to 32.83% (Figure 5e,f). Furthermore, we evaluated the pretreatment duration of 60 samples using both the conventional and ASAG563 methods. The traditional method [41,42] demanded an amount of 460 min for the process, whereas the ASAG563 method achieved the same in merely 243 min. This comparison reveals that the ASAG563 method has increased the efficiency of the pretreatment process by 50%. In summary, the ASAG563 purification column demonstrates broader applicability for TCTs and shows a notable advantage in the removal of pigments and impurities; it simplified analyst manipulation with a consequent decrease in quantification errors and analysis time.

### 3.7. Application of ASAG563 in Maize for Feedstuff Samples

**Figure 6:**
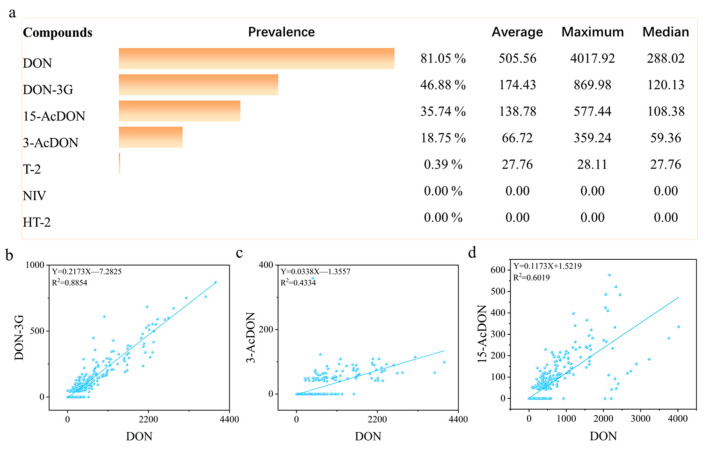
Assessment of contamination of TCTs in maize for feedstuff samples: (**a**) summary of TCTs contamination in maize for feedstuffs; (**b**) correlation of DON with DON-3G; (**c**) correlation of DON with 3-AcDON; (**d**) correlation of DON with 15-AcDON.

We analyzed 512 samples of maize for feedstuffs randomly collected from Northeast China using the developed ASAG563 purification column. The distribution of contamination is detailed in Table S7 and Figure 6. The detection results revealed the presence of five types of TCTs. Among these, DON was the most severely contaminated, with 81.05% of the maize samples testing positive for DON and average and median concentrations of 288.02 µg/kg and 505.56 µg/kg, respectively; notably, none of the samples exceeded the maximum regulation level of 5000 µg/kg [43]. The contamination of DON-3G was the next highest, detected in 46.88% of all samples, with average and median concentrations of 174.43 µg/kg and 120.13 µg/kg, respectively. The detection rates for 15-AcDON and 3-AcDON were relatively low, at 35.74% and 18.75%, respectively; however, their average and median concentrations were both greater than 59.36 µg/kg (Table S7 and Figure 6a). Only one sample tested positive for T-2 toxin, which is negligible regarding sample contamination. NIV nor HT-2 toxin was detected in any of the samples.

Furthermore, we analyzed the correlation between DON and the other three TCTs (Figure 6b–d) and found that DON-3G, 3-AcDON, and 15-AcDON were positively correlated with DON, with correlation coefficients (R^2^) of 0.8854, 0.4344, and 0.6019, respectively. This indicates that as the concentration of DON increases, the probability of contamination by these three TCTs also rises accordingly. In summary, the primary contaminants in the maize for feedstuff samples were DON, DON-3G, 3-AcDON, and 15-AcDON, all of which were found at high levels. Although none of the samples exceeded the maximum limits, continuous monitoring is essential due to climate change, evolving fungal populations, and the growing demand for global food security.

## 4. Conclusions

This study presents the development of a novel integrated multifunctional pretreatment column (ASAG563) designed to integrate extraction, purification, and filtration for detecting TCTs in cereal and feed matrices. The ASAG563 column addresses critical limitations of existing methods, such as operational complexity, high material consumption, and inadequate removal of impurities. The systematic evaluation demonstrated its superior performance, with recoveries ranging from 80.8% to 117.8% across various matrices, quantification limits as low as 2.02 µg/kg, and significantly reduced matrix effects. Compared to commercial products, the ASAG563 column achieved a 50% improvement in pretreatment efficiency while minimizing environmental impact. The innovation lies in the column’s integration of advanced sorbents (100 mg C18 + 250 mg MAX + 100 mg MCX + 50 mg POLY + 50 mg GCB) and optimized column designs, ensuring high selectivity and efficiency in removing pigments, lipids, and other matrix interferences. Its environmentally friendly design and streamlined workflow reduce material costs and processing time and decrease quantification errors, making it a valuable tool for large-scale applications in food safety and quality control.

The study’s findings underline the urgent need for enhanced monitoring of TCTs, particularly DON and its derivatives, in contaminated maize. Future research could explore extending the column’s applicability to other mycotoxin types and further optimizing sorbent compositions to enhance versatility. By providing a robust, efficient, and eco-friendly pretreatment solution, this study sets a new benchmark for analytical methods in food safety, with broad implications for regulatory frameworks and agricultural practices globally.

## Figures and Tables

**Table 1:** Analytical performance of ASAG563.

	Background of Column	Recovery of Column	
	C (μg/kg)	Recovery (%)	RSD (%)
NIV	<LOD	97.4	3.5
DON	<LOD	99.4	2.4
DON-3G	<LOD	88.4	3.8
3-AcDON	<LOD	99.7	3.0
15-AcDON	<LOD	100.8	2.5
T-2	<LOD	95.8	2.8
HT-2	<LOD	100.0	3.4

**Table 2:** Spiked concentration (C), recovery (REC), matrix effect (ME), intra-day precision (RSD_r_), and inter-day precision (RSD_R_) of seven TCTs (n = 3).

			Intra-Day									Inter-Day		
			Low Concentration Level			Medium Concentration Level			High Concentration Level			Medium Concentration Level		
Matrix	TCTs	ME (%)	C (μg/kg)	REC (%)	RSD_r_ (%)	C (μg/kg)	REC (%)	RSD_r_ (%)	C (μg/kg)	REC (%)	RSD_r_ (%)	C (μg/kg)	REC (%)	RSD_R_ (%)
Maize for feedstuff	NIV	103.0	800	92.2	0.3	1600	92.4	2.9	3200	92.4	2.9	1600	92.9	1.2
	DON	102.3	600	103.9	0.6	1200	100.0	1.4	2400	100.2	1.5	1200	99.1	2.0
	DON-3G	96.7	100	94.4	5.1	200	99.6	2.0	400	117.8	5.9	200	96.8	2.6
	3-AcDON	105.7	160	109.8	2.4	320	106.3	2.0	640	105.7	1.3	320	104.8	1.2
	15-AcDON	94.7	80	105.8	1.2	160	103.4	0.3	320	104.8	1.4	160	104.6	3.5
	T-2	105.8	8	113.8	2.5	16	103.6	1.3	32	100.2	6.8	16	105.0	1.9
	HT-2	103.0	40	108.8	5.6	80	104.9	0.1	160	96.1	2.8	80	105.8	0.9
DDGS	NIV	100.7	1600	90.2	0.2	3200	84.6	3.1	6400	80.8	1.3	3200	85.1	3.4
	DON	106.5	1200	102.4	1.2	2400	93.6	1.1	4800	85.4	0.8	2400	91.6	2.2
	DON-3G	132.8	200	94.3	3.0	400	86.0	3.1	800	78.9	4.8	400	84.3	1.7
	3-AcDON	100.8	320	110.2	0.8	640	104.5	0.8	1280	96.8	0.6	640	105.5	2.0
	15-AcDON	81.0	160	107.1	4.1	320	97.9	1.8	640	93.3	1.0	320	96.5	2.8
	T-2	105.0	16	111.5	3.1	32	112.0	1.4	64	101.7	0.4	32	110.6	1.4
	HT-2	106.6	80	100.5	3.7	160	109.2	0.5	320	102.1	2.6	160	109.0	3.3

## Data Availability

The original contributions presented in this study are included in the article/Supplementary Materials. Further inquiries can be directed to the corresponding authors.

## Supplementary Materials

The following supporting information can be downloaded at: https://www.mdpi.com/article/10.3390/foods14091466/s1, Figure S1. Total ion chromatography (TIC) of sorbents; Figure S2. Adsorption behavior of seven TCTs on filter membranes and sieve plates; Table S1. Concentrations of external standards; Table S2. Concentrations of internal standards; Table S3. MS/MS parameters of 17 mycotoxins; Table S4. Database on the purification capacity of individual sorbent; Table S5. Verification results of linear regression equations, correlation coefficients (R^2^), and linear ranges; Table S6. Results of the certified reference material of DON in maize flour pretreated by ASAG563; Table S7. Contamination of TCTs in maize for feedstuffs.
