# Supplementary material for: Development of an Integrated Multifunctional Column for Rapid Pretreatment and Determination of Trichothecenes in Cereals and Feeds with HPLC-MS/MS
Source: Foods. 2025 Apr 23;14(9):1466. doi: 10.3390/foods14091466 (PMC12071380; doi:10.3390/foods14091466)
Supplement: Supplementary file 1 [file foods-14-01466-s001.zip › foods-3574764-supplementary.pdf]

supplementary materials for

Development of an Integrated Multifunctional Column for Rapid Pretreatment and Determination of Trichothecenes in Cereals and Feeds with HPLC-MS/MS

Sisi Liu [1,2,†], Yu Wu [2,†], Tongtong Liu [2], Jin Ye [2,*], Li Li [2], Xiao Guan [1] and Songxue Wang [2,*][1] School of Health Science and Engineering, University of Shanghai for Science and Technology, Shanghai 200093, China; s15907925623@126.com (S.L.); gnxo@163.com (X.G.)

[2] NFSRA Key Laboratory of Grain and Oil Quality and Safety, Academy of National Food and Strategic Reserves Administration, Beijing 100037, China; wyu@ags.ac.cn (Y.W.); wy13270353@163.com (T.L.); ll@ags.ac.cn (L.L.)* Correspondence: yj@ags.ac.cn;wsx@ags.ac.cn.

[†] These authors have contributed equally to this work and share first authorship.

Supporting Caption

Figure S1. Total ion chromatography (TIC) of sorbents.

Figure S2. Adsorption behavior of seven TCTs on filter membranes and sieve plates.

Table S1. Concentrations of external standards.

Table S2. Concentrations of internal standards.

Table S3. MS/MS parameters of 17 mycotoxins.

Table S4. Database on the purification capacity of individual sorbent.

Table S5. Verification results of linear regression equations, correlation coefficients (R^2), and linear ranges.

Table S6. Results of the certified reference material of DON in maize flour pretreated by ASAG563.

Table S7. Contamination of TCTs in maize for feedstuffs.

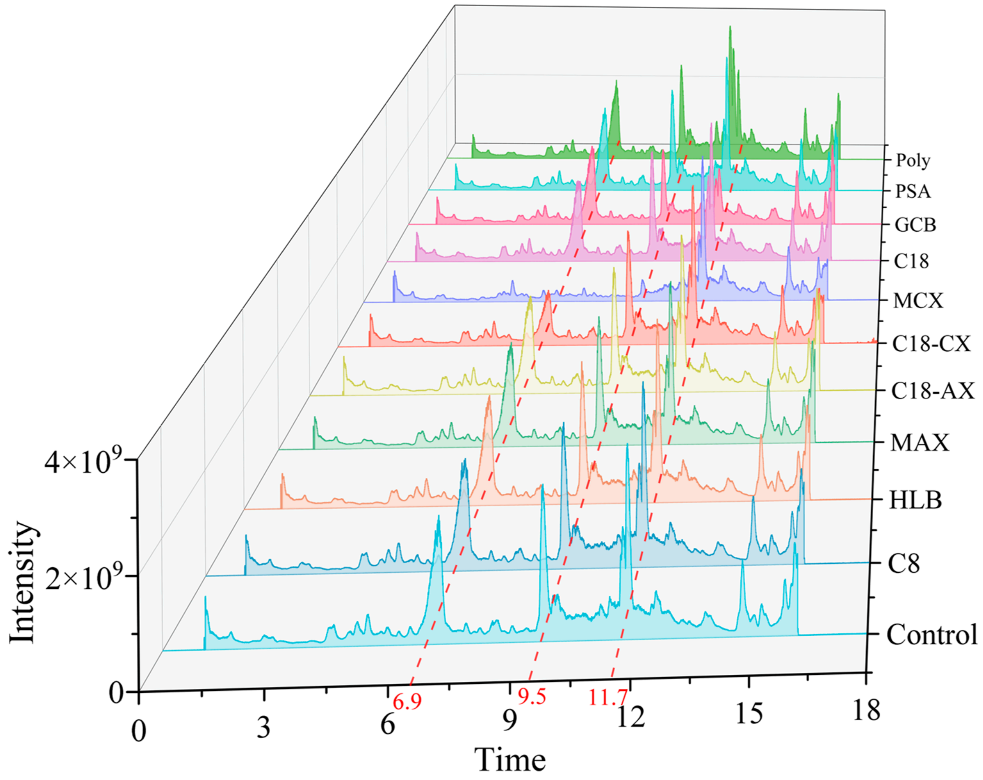

Figure S1. Total ion chromatography (TIC) of sorbents (the dotted lines at 6.9, 9.54 and 11.7 min indicate the retention times of the interference peaks)

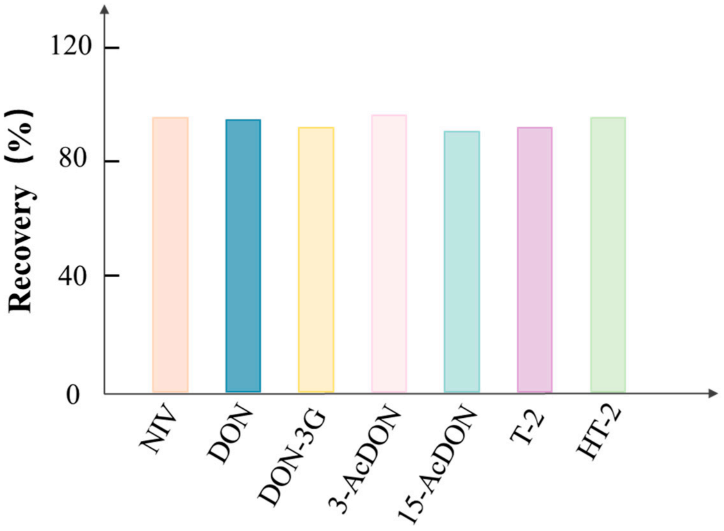

Figure S2. Adsorption behavior of seven TCTs on filter membranes and sieve plates

Table S1. Concentrations of external standards.

Type of Mycotoxins	Stocked Solution (μg/L)	High spiked concentration level (μg/kg)	Medium spiked concentration level (μg/kg)	Low spiked concentration level (μg/kg)
NIV	20000	3200	1600	800

DON	15000	2400	1200	600
DON-3G	2500	400	200	100
3-AcDON	4000	640	320	160
15-AcDON	2000	320	160	80
T-2	100	32	16	8
HT-2	100	160	80	40
AFB1	100	16	8	4
AFB2	100	16	8	4
AFG1	2000	16	8	4
AFG2	1000	16	8	4
FB1	1000	320	160	80
FB2	200	160	80	40
FB3	1000	160	80	40
ZEN	2000	320	160	80
OTA	200	32	16	8
ST	100	16	8	4

Table S2. Concentrations of internal standards.

Type of Mycotoxins	Stocked Solution (μg/L)
^{13}[C]NIV	2650
^{13}[C]DON	2000
^{13}[C]3-AcDON	1000
^{13}[C]T-2	50
^{13}[C]HT-2	125

Table S3. MS/MS parameters of 17 mycotoxins.

Compounds	ESI Mode	Retention Time(min)	Translations (m/z)	Declusterring Potential (V)	Collision Energy (eV)	Collision Cell Exit Potential (V)
NIV	-	1.70	357.2/311.0*, 357.2/281.0	-40, -40	-13, -13	-13, -13
[$^{13}C_{15}$]-NIV	-	1.70	372.1/295.0	-40	-13	-13
DON	+	2.63	297.3/231.3*, 297.3/249.3	75, 75	17, 15	7, 7
[$^{13}C_{15}$]-DON	+	2.63	312.2/263.1	75	15	7
DON-3G	+	2.20	476.1/297*, 476.1/249.0	50, 50	19, 31	14, 16
3-AcDON	+	6.50	339.2/231.1*, 339.2/213.1	75, 75	18, 22	13, 13
[$^{13}C_{17}$]-3-AcDON	+	6.50	356.2/245.1	75	18	13
15-AcDON	+	6.40	356.2/137*,356.2/321.0	20, 20	23, 19	13, 13
T-2	+	11.00	484.2/305.1*, 484.2/185.0	20, 20	19, 27	12, 14
[$^{13}C_{24}$]-T-2	+	11.00	508.2/322.1	20	19	12
HT-2	+	10.11	442.1/263.1*, 442.1/215.0	40, 40	17, 19	10, 13
[$^{13}C_{22}$]-HT-2	+	10.11	464.1/278.1	40	17	10
AFB1	+	10.07	313.1/285*, 313.1/241.1	105, 105	30, 50	10, 10

[$^{13}C_{17}$]-AFB1	+	10.07	330.1/301.1	105	30	10
AFB2	+	9.54	315.0/287.0*, 315/259.0	80, 80	34, 38	13, 13
[$^{13}C_{17}$]-AFB2	+	9.54	332.1/30.1	80	34	13
AFG1	+	9.72	329..1/243*, 329.1/311.0	100, 100	35, 30	11, 8.5
[$^{13}C_{17}$]-AFG1	+	9.72	346.1/257.1	100	35	11
AFG2	+	8.65	331.1/245*, 331.1/285	100, 100	40, 37	10, 10
[$^{13}C_{17}$]-AFG2	+	8.65	348.1/259.1	90	40	10
FB1	+	9.87	722.5/334.3, 722.5/352.3	40, 40	54, 49	22, 22
[$^{13}C_{34}$]-FB1	+	9.87	756.5/356.3	40	54	22
FB2	+	10.23	706.5/336.3*, 706.5/318.2	30, 30	50, 43	22, 15
[$^{13}C_{34}$]-FB2	+	10.23	740.5/358.3	30	50	22
FB3	+	10.05	706.5/336.3*, 706.5/318.2	30, 30	50, 43	22, 15
[$^{13}C_{34}$]-FB3	+	10.05	740.5/358.3	30	50	22
ZEN	-	11.80	317/175*, 317/131	-80, -80	-25, -40	-7, -15
[$^{13}C_{18}$]-ZEN	-	11.80	335.1/185.0	-80	-25	-7
OTA	+	11.50	404.0/239.0*, 404.0/358.0	30, 30	26, 30	13, 13
[$^{13}C_{20}$]-OTA	+	11.50	424.1/250.0	30	30	13
ST	+	12.40	325.1/309.9*, 325.1/280.9	90, 90	35, 51	12, 18
[$^{13}C_{18}$]- ST	+	12.40	343.1/327.0	90	35	12

* Represents quantitative ions

Table S4. Database on the purification capacity of individual sorbent.

Name	Characteristics	Type of Impurities	Applicable Mycotoxin Species	Remove Pigment	Remove Visible Impurities	Remove Interfering Peak
C8	Octylsilane bonded phases	Adsorption of non-polar compounds and lipid impurities	17	slight	slight	No significant removal
HLB	Hydrophilic-ester balance, hydrophilic and hydrophobic functional groups on the surface at the same time	Balanced adsorption of various polar and non-polar impurities.	12(not ZEN/OTA/ST/AFB1/AFB2)	moderate	slight	No significant removal
MAX	Hybrid strong anion exchange	Adsorption of acidic impurities	17	slight	slight	No significant removal
C18-AX	C18-modified with both anion exchange and C18 groups	Adsorption of lipid and acidic impurities	17	slight	moderate	No significant removal
C18-CX	C18-modified with both cation exchange and C18 groups	Adsorption of lipid and alkaline impurities	17	slight	moderate	Removal 6.9min
MCX	Hybrid strong cation exchange	Adsorption of alkaline impurities	14(not FBS)	slight	slight	significant remove 6.9 min and 9.5 min
C18	Octylsilane-bonded phases	Adsorption of non-polar compounds and lipid impurities	17	moderate	moderate	No significant removal
GCB	carbon graphitization	Adsorption of laminar structure impurities and pigments	10(not ZEN\OTA\ST\AFS)	strong	strong	significant removal 11.7 min

PSA	Primary and secondary amine groups	Adsorption to remove organic acids, pigments, metal ions and phenolic impurities	14(not FBS)	slight	slight	No significant removal
POLY	High Nitrogen Loading Weakly Cationic Long Chain Polymer Fillers	Effective in removing grease	17	slight	slight	significant removal 11.7 min

Table S5. Verification results of linear regression equations, correlation coefficients (R^2), and linear ranges.

TCTs	LOQ(μg/kg)	Regression Equation	R^2	Range(μg/L)
NIV	48.41	Y=21200X-28300	0.9999	50.0-2000.0
DON	39.49	Y=0.00547X-0.027	0.9995	37.5-1500.0
DON-3G	19.17	Y=62900X-50700	0.9990	6.25-250.0
3-AcDON	11.16	Y=157000X-119000	0.9992	10.0-400.0
15-AcDON	5.67	Y=217000X-53700	0.9991	5.0-200.0
T-2	2.02	Y=1520000X-283000	0.9999	0.5-20.0
HT-2	11.92	Y=153000X-128000	0.9996	2.5-100.0

Table S6. Results of the certified reference material of DON in maize flour pretreated by ASAG563.

	DON (μg/kg)
Assigned Value	990.0
Range for |z|≤2	130.0
YMDD-1	1015.9
YMDD-2	1052.0
YMDD-3	1062.2
YMDD-4	1022.8
YMDD-5	1030.8
YMDD-6	999.0
AVE	1030.5
RSD(%)	2.3

Table S7. Contamination of TCTs in maize for feedstuffs.

Compounds	Prevalence	Average	Maximum	Median
DON	81.05 %	505.56	4017.92	288.02
DON-3G	46.88 %	174.43	869.98	120.13
15-AcDON	35.74 %	138.78	577.44	108.38
3-AcDON	18.75 %	66.72	359.24	59.36
T-2	0.39 %	27.76	28.11	27.76
NIV	0.00 %	0.00	0.00	0.00
HT-2	0.00 %	0.00	0.00	0.00
